# Increased Risk of Ulcerative Colitis in Patients with Periodontal Disease: A Nationwide Population-Based Cohort Study

**DOI:** 10.3390/ijerph15112602

**Published:** 2018-11-21

**Authors:** Chien-Yu Lin, Kuo-Sen Tseng, Jui-Ming Liu, Heng-Chang Chuang, Chi-Hone Lien, Yi-Chih Chen, Chun-Ying Lai, Cheng-Ping Yu, Ren-Jun Hsu

**Affiliations:** 1 Department of Pediatrics, Hsinchu MacKay Memorial Hospital, Hsinchu city 300, Taiwan; mmhped.lin@gmail.com (C.-Y.L.); 4976@mmh.org.tw (C.-H.L.); marion4352546@gmail.com (Y.-C.C.); 2 Division of Rheumatology, Department of Internal Medicine, Taoyuan General Hospital, Ministry of Health and Welfare, Taoyuan 330, Taiwan; guosentseng@gmail.com; 3 Division of Urology, Department of Surgery, Taoyuan General Hospital, Ministry of Health and Welfare, Taoyuan 330, Taiwan; mento1218@gmail.com (J.-M.L.); chuang20110617@yahoo.com.tw (H.-C.C.); 4 Graduate Institute of Life Sciences, National Defense Medical Center, Taipei 114, Taiwan; 5 Division of Gastroenterology, Department of Internal Medicine, Taoyuan General Hospital, Ministry of Health and Welfare, Taoyuan 330, Taiwan; jackylai423@gmail.com; 6 Biobank Management Center of the Tri-Service General Hospital, National Defense Medical Center, Taipei 114, Taiwan; cpyupath@gmail.com; 7 Department of Pathology and Graduate Institute of Pathology and Parasitology, The Tri-Service General Hospital, National Defense Medical Center, Taipei 114, Taiwan

**Keywords:** Crohn’s disease, inflammatory bowel disease, periodontal disease, ulcerative colitis, nationwide, national health insurance research database

## Abstract

Both periodontal disease (PD) and inflammatory bowel disease (IBD), including Crohn’s disease (CD) and ulcerative colitis (UC), are important diseases of the alimentary tract. Microbiome and immune-mediated inflammatory processes play important roles in these diseases. An association between PD and IBD may exist. This study investigated the risk of IBD in patients with PD. This study used data from the National Health Insurance Research Database of Taiwan from 1996 to 2013. A total of 27,041 patients with PD were enrolled as a study group, and 108,149 patients without PD were selected as the control group after matching by gender, age, insured region, urbanization, and income with a 1:4 ratio. Cox proportional hazards regression was used to calculate the risk of IBD. Of the 135,190 participants enrolled in this study, 5392 (4%) with newly diagnosed IBD were identified. The overall incidence of subsequent IBD was similar in both groups (3.8% vs. 4%, adjusted hazard ratio (aHR) = 1.01, 95% confidence interval (CI): 0.94–1.08). However, an increased risk of UC in the PD group was found after adjusting confounding factors (aHR: 1.56, 95% CI: 1.13–2.15; *p* < 0.05). This study demonstrated that patients with PD had approximately one-half higher risk of subsequent UC. Further studies are warranted to elucidate the relationship between PD and UC.

## 1. Introduction

Inflammatory bowel disease (IBD) is a chronic inflammatory disease involving the gastrointestinal tract. As globalization and rapid economic development have been observed worldwide, the incidence of IBD is increasing [1]. Approximately 1.4 million Americans are affected by IBD with the peak onset from 15 to 30 years of age [2]. In addition, 2.5–3 million people in Europe are estimated to be affected by IBD, with a direct healthcare cost of 4.6–5.6 billion Euros per year [3]. IBD comprises two diseases: ulcerative colitis (UC) and Crohn’s disease (CD), but the clinical manifestations and pathophysiology are different in these two conditions [4]. The pathogenesis is multifactorial. As whole genome studies are advancing, mounting evidence emphasizes the important role of host–microbe interactions in a genetically susceptible host [4,5]. Multiple genes regulate inflammatory pathways that are associated with IBD, such as genomic regions containing nucleotide-binding oligomerization domain 2, autophagy genes, and the interleukin (IL)-23 and T helper 17 (Th17) cell pathways [4]. Furthermore, the gastrointestinal tract is obligately not bacteria free, and inappropriate inflammatory responses triggered by gut microbiota induce chronic intestinal inflammation and cause IBD [4,5,6,7,8]. Dysbiosis will cause intestinal inflammation, impair barrier function, stimulate specific cytokine release, interfere nutrients absorption, activate toxins, genotoxins, and mutagens [7]. Several bacteria have been found that are involved in the pathogenesis of IBD and increase the risks of IBD, such as Clostridium, Staphylococcus, and the genera Bacteroides, but the primary etiologic agents are unknown [6,7]. The entire mechanism of host–microbiota reactions and subsequent inflammatory processes remains largely unclear.

Periodontal disease (PD) is an inflammatory disease involving the structures surrounding the teeth [9]. Of the global population, 20–50% has PD, and impairment of life quality is common [9,10,11]. Similarly, colonization and growth of microorganisms cause local and systemic inflammatory responses and destroy the gums, periodontal ligament, or alveolar bone [10]. The etiological factors and pathogenesis of PD are multifactorial, and developing PD involves both local and systemic influences [12,13]. *Porphyromonas gingivalis*, *Aggregatibacter actinomycetemcomitans*, *Bacteroides forsythus*, *Prevotella intermedia*, *Peptostreptococcus micros*, and *Fusobacterium nucleatum* are strongly associated with PD [12]. Microbe colonization and plaque buildup result in chronic local inflammation, breakdown of barrier, and subsequent damage of surrounding gum tissue [12]. Patients who smoke tobacco and have diabetes mellitus (DM) also have a higher risk of PD [12]. Furthermore, alterations of cytokines in saliva or serum are observed in patients with PD, including IL-1, IL-6, IL-10, IL-17A, IL-17F, IL-22, IL-25, IL-33, tumor necrosis factor-alpha, and interferon-gamma [14,15]. The pathophysiology of PD is complex and local inflammation caused by periodontal colonizing microorganisms may induce subsequent systemic influences [12,13]. Increased risks of systemic diseases following PD have been reported [10]. Patients with PD have a 19% increase in the risk of cardiovascular diseases, and individuals with type 2 diabetes with severe forms of PD have 3.2 times greater mortality risk [10]. Effective disease management requires aggressive local treatment, modification of associated risk factors, and prevention of long-term unfavorable systemic diseases [10,11,12].

Several similarities have been observed in IBD and PD. They are both chronic mucosal inflammatory diseases of the alimentary tract. Microbiome and immune-mediated inflammatory processes play important roles in both diseases, and both have systemic impact. Cytokine changes were observed in both diseases and Th1/Th17-related pro-inflammatory cytokines were also involved in the pathophysiology of these two diseases [15,16]. They also share similar environmental and lifestyle-related risk factors [4,9]. An association between these two diseases may exist. In patients with IBD, higher comorbid risk of PD is noted compared with individuals without IBD [17,18,19,20]. The increased risk of PD in patients with IBD was well known and a systematic review showed a significant increased risk of PD in patients with IBD (332 more patients per 1000 patients) [17]. Patients with IBD also had more severe PD (higher score for the Decayed-Missing-Filled Teeth index or DMFT) [17]. However, the complete mechanism of the increased risk is not fully understood, and the interaction between these two diseases may be bidirectional. Therefore, we conducted this retrospective large-scaled nationwide study to investigate the risk of subsequent IBD in patients with PD.

## 2. Materials and Methods

### 2.1. Data Source and Collection

The National Health Insurance program of Taiwan is a nationwide medical insurance system that covered 99.5% of Taiwan’s 23 million residents [21]. Therefore, data from this National Health Insurance program is highly representative [22]. The National Health Insurance Research Database (NHIRD) was categorized and sorted by the International Classification of Diseases, 9th revision, Clinical Modification (ICD-9-CM) coding system. Details regarding patient diagnosis and treatment-related information are included in the database [23]. Longitudinal Health Insurance Database 2000 (LHID2000) is a subset of data from the NHIRD, and it is composed of one million randomly selected individuals from the NHIRD in 2000. This cohort study used LHID2000. This study was approved by the Institutional Review Board of the Tri-Service General Hospital (approval number: TSGHIRB NO B-104-21).

### 2.2. Study Population

**Figure 1:**
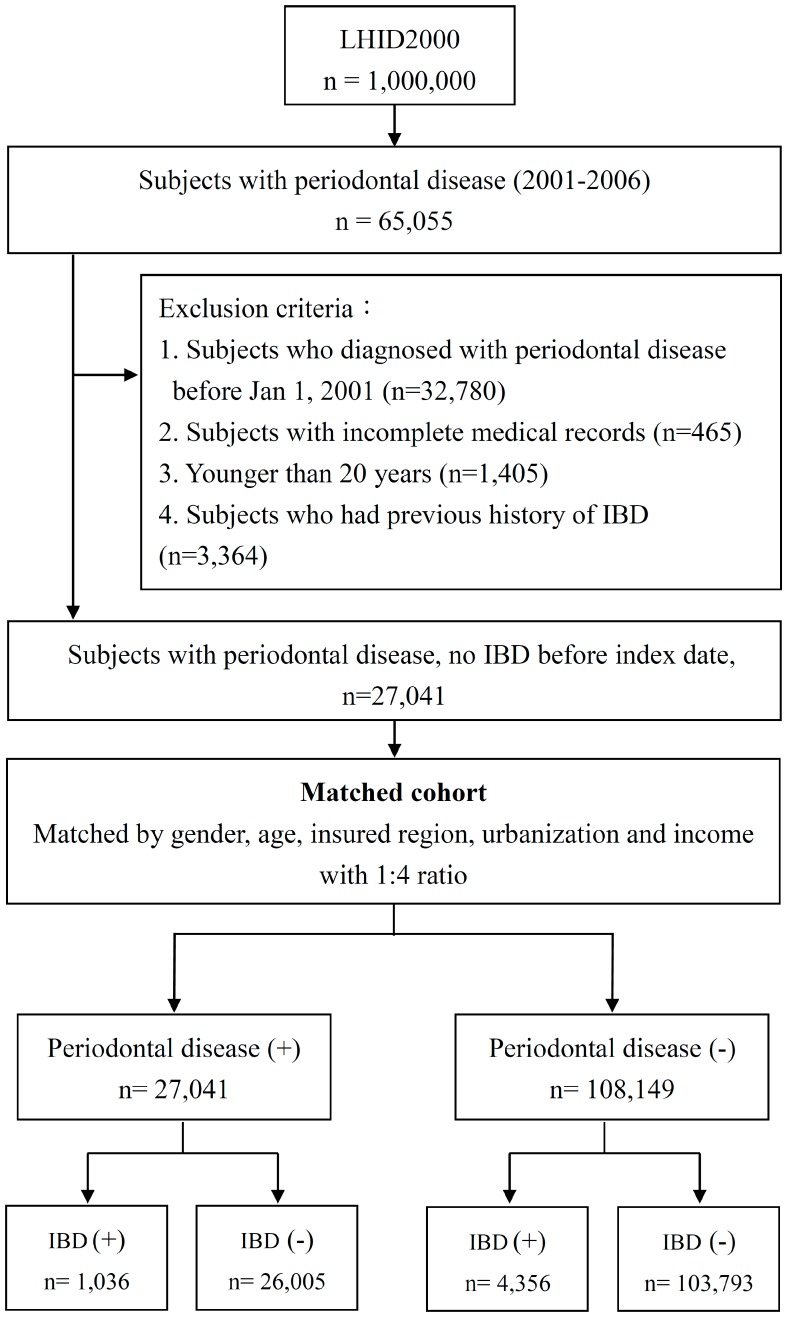
The flow chart for enrollment of study cohorts. LHID: Longitudinal Health Insurance Database; IBD: inflammatory bowel disease.

The flow chart of enrollment is shown in Figure 1. Participants were selected by using the LHID2000 between January 1996 and December 2013. From 2001 to 2006, patients with PD were identified. The exclusion criteria included (1) history of PD before 2001, (2) incomplete medical records, (3) younger than 20 years, and (4) previous history of autoimmune disease. The index date referred to the date of diagnosis of PD (ICD-9-CM: 523.3, 523.4, and 523.5). The diagnosis of PD was made by licensed dentists. Individuals with PD were enrolled as the PD group. Furthermore, for each patient in the PD group, a matched control was identified with the same gender, age, insured region, urbanization, and income with a 1:4 ratio. We tracked both groups to identify individuals newly diagnosed with IBD. IBD was defined as CD (ICD-9-CM: 555.0, 555.1, 555.2, and 555.9) and UC (ICD-9-CM: 556.0, 556.6, 556.8, and 556.9).

### 2.3. Study Outcomes and Covariates

Each patient was tracked for a 13-year period starting with their index date to identify those who were newly diagnosed with IBD. The primary outcome was newly diagnosed IBD, which was mainly determined by a gastroenterologist. IBD is a catastrophic illness in Taiwan and proof of diagnoses has to be reviewed by special committee. The diagnosis of IBD was based on medical history, clinical evaluation, endoscopic findings, and histological findings [24,25]. Censoring was defined as death, the date of diagnosis of IBD, or the end of the follow-up period on 31 December 2013. Medical and demographic variables for both groups were also extracted and analyzed, including age, monthly income, geographic area of residence, urbanization level of residence, and comorbidities. Age was divided into six groups based on 10-year intervals: 20 to 29 years, 30 to 39 years, 40 to 49 years, 50 to 59 years, and ≥60 years. The monthly income of the study population was recorded in New Taiwan Dollars (NTD) and categorized into four income levels: <NTD 20,000, NTD 20,000 to NTD 39,999, NTD 40,000 to NTD 59,999, and ≥NTD 60,000. The geographic regions in Taiwan were divided into four areas: northern region, central region, southern region, and other regions (eastern and outlying islands). The urbanized level of residence in Taiwan was classified into four categories. Comorbid diseases included DM (ICD-9-CM: 250), hypertension (ICD-9-CM: 401–405), hyperlipidemia (ICD-9-CM: 272), coronary artery disease (ICD-9-CM: 410–414), stroke (ICD-9-CM: 430–438), alcoholism (ICD-9-CM: 291, 303, 305.00–305.03, 571.1, 571.2, 571.3, 790.3, A215, and V11.3), obesity (ICD-9-CM: 278), and tobacco use disorder (ICD-9-CM: 305.1, 491.0, 491.2, 492.8, 496, 523.6, 649.0, 989.84, and V15.82). All covariates and comorbidities of both groups were explored and analyzed.

### 2.4. Statistical Analysis

Student’s *t*-test and chi-square test were used to analyze and compare the categorical demographic characteristics including age, income, geographic area of residence, level of urbanization of residence, and comorbidities of both groups. The incidence rate for IBD for both groups was tracked and calculated. Cox proportional hazards regressions were performed to evaluate the relationship between PD and subsequent IBD, and the hazard ratio (HR) was calculated with 95% confidence interval (CI). Further adjustment for potential confounders (age, gender, income, geographic area of residence, level of urbanization of residence, and comorbidities) was performed in all models, and adjusted HR (aHR) was calculated. A two-sided *p*-value < 0.05 was considered statistically significant. The statistical analyses were performed using the SPSS software version 19.0 (SPSS Inc., Chicago, IL, USA), and data were managed with Microsoft^®^ SQL Server^®^ 2008 software (Microsoft Unternehmen, Redmond, DC, USA).

## 3. Results

A total of 27,041 patients were identified with PD but without a previous history of autoimmune disease (Figure 1). After matching for age and gender with 1:4 ratio, 108,149 patients without PD were enrolled in the control group. In total, 135,190 patients were enrolled in this study and followed to investigate the incidence of IBD.

Demographic data are shown in Table 1. There were no significant differences in age and gender. Most patients had low income and resided in highly urbanized areas in northern Taiwan. Patients with PD had a higher incidence of comorbid diseases, including DM, hypertension, hyperlipidemia, coronary arterial disease (CAD), stroke, obesity, and tobacco use disorder.

**Figure 2:**
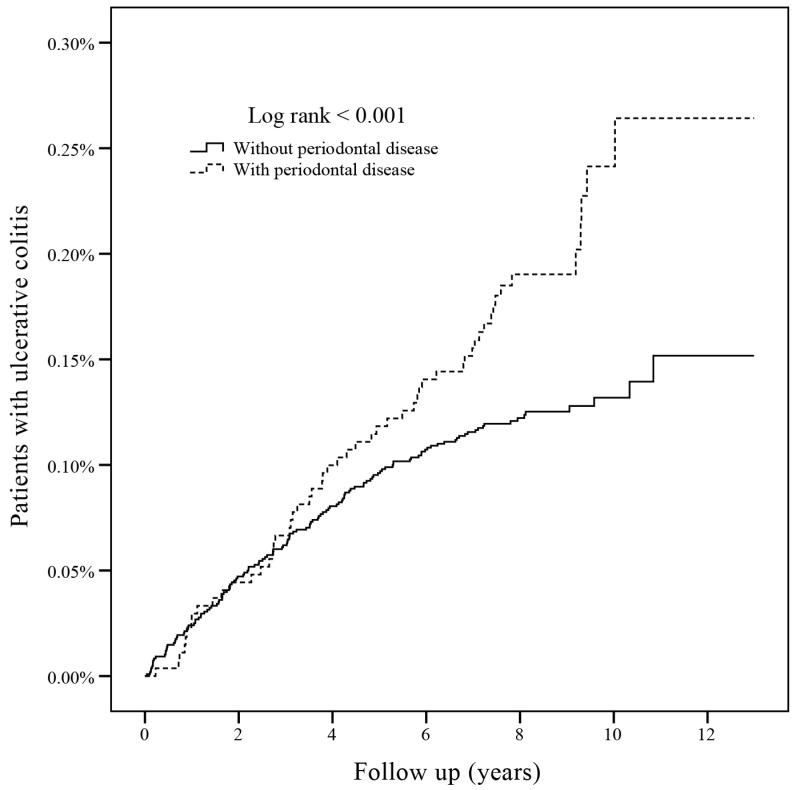
The Kaplan–Meier survival curve of ulcerative colitis in both groups.

During the 13-year follow-up period, 5392 (3.99%), patients had IBD, and the incidence of IBD was similar between the PD and non-PD group (3.83% vs. 4.03%, aHR = 1.01, 95% CI: 0.94–1.08). The mean duration of follow-up was 8.47 ± 1.70 (mean ± standard deviation) years (PD group: 8.53 ± 1.60 years, control group: 8.47 ± 1.72 years). The period from enrollment to diagnosis of IBD was 4.11 ± 2.58 years in PD group and 3.22 ± 2.74 years in control group. Specifically, patients with PD had significantly higher subsequent risk of UC (0.20% vs. 0.12%, crude HR: 1.61, 95% CI: 1.17–2.20, *p* < 0.05). After adjusting for confounding factors, the aHR was 1.56 (95% CI: 1.13–2.15, *p* < 0.05) with statistical significance listed in Table 2. The incidence rate was 0.23 per 1000 person-years for the PD group and 0.15 per 1000 person-years for the control group. The Kaplan–Meier survival curve is plotted in Figure 2 and demonstrates the incidence of UC in both groups. Cox proportional hazards regression was performed to evaluate the independent risk of subsequent UC, and the results are shown in Table 3. Regarding the gender of patients with UC, there was no difference between genders. High income was a risk factor for UC (aHR: 2.29, 95% CI: 1.28–4.11, *p* < 0.05). No significant associated risk factors were observed in comorbidities except for patients with tobacco use disorder had a lower risk of UC (aHR: 0.47% CI: 0.27–0.81, *p* < 0.05).

## 4. Discussion

To the best of our knowledge, this is the first study specifically investigating the relationship between PD and IBD. Both PD and IBD are chronic inflammatory mucosal diseases of the alimentary tract. Higher risk of PD in patients with IBD was well known but whether the increased risk was bidirectional remained unclear. In this large cohort study, 27,041 patients with PD had approximately one-half increased risk of subsequent UC during a 13-year follow-up period compared with the control group. The association between PD and UC may be bidirectional and further studies are required to elucidate the underpinning pathophysiology.

The increased risk for UC observed in patients with PD may result from several factors, including mucosal microbiota, host inflammatory responses, and the interaction of microbe–host immunity [26,27]. In patients with PD, microbe colonization and plaque buildup result in chronic local inflammation and subsequent damage of surrounding gum tissue [26]. However, long-term host responses against the bacterial challenge from the dental plaque will cause systemic inflammatory cascade and result in higher risk of systemic diseases. Mounting evidence has shown a higher risk of cardiovascular diseases, diabetes, rheumatoid arthritis, preeclampsia, and preterm birth [10,13,28]. Alterations of cytokines are detected in patients with PD, including IL-1, IL-6, IL-10, IL-17A, IL-17F, IL-22, IL-25, IL-33, tumor necrosis factor-alpha, and interferon-gamma [14,15,29,30,31,32]. Salivary cytokines may be considered a biomarker of PD [31]. There is still some evidence showing that PD is a local inflammatory disease with systemic influences via immune-mediated responses. IBD is undoubtedly an immune-mediated disease and may be affected by the inflammatory responses induced by PD. Marked cytokine alterations are also observed in patients with IBD [16,32,33]. We found that PD increases the risk of UC, but not CD.

Patients with IBD had a higher risk of PD and more severe PD (higher DMFT index) in previous studies [14,15,16,27,34,35,36]. The odds ratio was 4.55, and the mean difference in DMFT index was 3.85 in the systematic review and meta-analysis published in 2017 [17]. There are substantial and consistent studies supporting the higher risk of PD in patients with IBD, and the reported odds ratio ranged from 2 to 7 [18]. The link between IBD and PD is thought to originate from both environmental and host factors. These two diseases are both disproportionate chronic mucosal inflammatory diseases and share similar environmental and lifestyle risk factors [18]. Expression of the HLA-B27 haplotype and IL-10 deficiency are associated with the link between PD and IBD [15,16]. Neutrophil recruitment occurs after challenge by mucosal microbiome, and humoral immune-mediated responses follow. Th1/Th17-related pro-inflammatory cytokines are also involved in the pathophysiology of these two diseases [15,16]. Increased risk of PD in patients with IBD is clear, but the complex immunological responses may be bidirectional. Our study finds an increased risk of UC in patients with PD and fills the gap in the research on this topic. Further studies are required to confirm our findings and elucidate the detailed pathophysiological mechanisms.

Generally, IBD compromises two categories of inflammatory diseases: UC and CD. They have many similarities, but they also have individually unique aspects [4,5,35]. They have different clinical manifestations, common locations, disease courses, prognosis, and treatment. In view of IBD and PD, although both UC and CD are associated with PD, the reported risk of PD is different between UC and CD. For example, in the case-controlled study conducted in Jordanians, patients with IBD had a significantly higher risk of PD, but the odds ratios were different in CD and UC (CD: odds ratio 4.9, 95% CI 1.8–13.2; UC: odds ratio 7.00, 95% CI 2.8–17.5) [37]. Patients with UC but not patients with CD had higher prevalence of deep ulcers in oral soft tissues than the non-IBD group [37]. Patients with UC had higher prevalence of PD than patients with CD and controls [19]. Higher prevalence of a variety of oral diseases has been observed in patients with UC [35]. Compared with CD, patients with UC had considerably worse oral health in most studies [17,35,38]. These two diseases have similar but distinct pathogenesis and clinical manifestations, and the response to oral microbiome may differ between the two subtypes of IBD [35]. UC may have stronger association with PD than CD, and our study strengthens the link between PD and UC.

Smoking has a different impact on the incidence of UC and CD. Smoking is an important environment risk factor and is known to have an impact on IBD. Interestingly, individuals who smoke have a higher risk of CD but a lower risk of UC [39]. Smoking may be protective against UC, and nicotine may have a role in the treatment of UC [40]. Both innate and adaptive immunity are affected by smoking, but the entire mechanism by which smoking exerts its impact on disease and the dichotomous effect in patients with CD and UC remains largely unclear. Our study found that patients who smoked had significantly lower risk of UC (aHR: 0.47, 95% CI: 0.27–0.81, Table 3), which is comparable with previous studies. Moreover, smoking may be a potent effect modifier in the interaction between PD and IBD. The reported risk of PD and high DMFT index different in smokers and non-smokers [19]. Further studies are warranted to investigate the influences of smoking.

The strength of our study is a nationwide database with broad coverage and findings that are representative of the general population. A 13-year follow-up of a large population made our results statistically and scientifically significant. However, there are some limitations. First, information regarding laboratory tests is not available in our database. It is valuable to measure the related cytokine alterations and microbiota to investigate the complete mechanism behind the observed association between PD and UC. Second, the severity of PD may affect the risk of IBD. The DMFT index and related local findings are incomplete in the current database. Patients with more severe PD may have a higher risk of IBD, but further studies are required to evaluate this hypothesis. Furthermore, the severity of IBD and treatment responses is not analyzed. The observed association may be affected by disease severity and prognosis.

## 5. Conclusions

In conclusion, this large-scale nationwide population-based study finds that patients with PD have approximately one-half higher risk of subsequent UC. Intestinal microbiota and host inflammatory response may contribute to the association. Further studies are warranted to investigate the underpinning mechanisms and elucidate the observed association between PD and UC.

## Figures and Tables

**Table 1:** Distribution of gender, age group and comorbidity in individuals with and without PD.

Variable	Number of Individuals		*p*-Value
	PD	Without PD	
	*n* = 27,041	*n* = 108,149	
Gender			
Female	13,068 (48.3%)	52,267 (48.3%)	0.995
Male	13,973 (51.7%)	55,882 (51.7%)	
Age Group			1
20–29	4524 (16.7%)	18,096 (16.7%)	
30–39	4567 (16.9%)	18,268 (16.9%)	
40–49	6557 (24.2%)	26,228 (24.3%)	
50–59	5746 (21.2%)	22,972 (21.2%)	
60–69	3131 (11.6%)	12,519 (11.6%)	
>69	2516 (9.3%)	10,066 (9.3%)	
Income Group			<0.001
<20000	17,429 (64.5%)	78,921 (73%)	
20000–39999	4964 (18.4%)	17,995 (16.6%)	
40000–59999	3032 (11.2%)	8090 (7.5%)	
≥60000	1616 (6%)	3143 (2.9%)	
Geography			<0.001
North	13,351 (49.4%)	55,469 (51.3%)	
Central	5467 (20.2%)	18,679 (17.3%)	
South	7570 (28%)	31,141 (28.8%)	
Other	653 (2.4%)	2860 (2.6%)	
Urbanization			<0.001
1 (highest)	14,199 (52.5%)	47,711 (44.1%)	
2	6613 (24.5%)	28,190 (26.1%)	
3	4543 (16.8%)	22,174 (20.5%)	
4 (lowest)	1686 (6.2%)	10,074 (9.3%)	
Comorbidity			
DM	6202 (22.9%)	18,593 (17.2%)	<0.001
Hypertension	10,262 (37.9%)	34,099 (31.5%)	<0.001
Hyperlipidemia	9678 (35.8%)	27,903 (25.8%)	<0.001
CAD	5418 (20%)	15,657 (14.5%)	<0.001
Stroke	3447 (12.7%)	10,738 (9.9%)	<0.001
Alcoholism	481 (1.8%)	2213 (2%)	<0.05
Obesity	436 (1.6%)	1292 (1.2%)	<0.001
Tobacco use disorder	4373 (16.2%)	12,026 (11.1%)	<0.001

PD, periodontal disease; DM, Diabetes mellitus; CAD, Coronary artery disease.

**Table 2:** The association between PD and IBD analyzed by Cox regression model.

	PD (*n* = 27,041)			Without PD (*n* = 108,149)			Crude	Adjusted
Disease	*n*	PY	IR	*n*	PY	IR	HR (95% CI)	HR * (95% CI)
**Inflammatory bowel disease (IBD)**	1036 (3.83%)	230,635	4.49	4356 (4.03%)	915,520	4.76	0.95 (0.88–1.01)	1.01 (0.94–1.08)
**Crohn’s disease**	985 (3.64%)	230,872	4.27	4235 (3.92%)	916,252	4.62	0.92 (0.86–0.99)	0.99 (0.92–1.06)
**Ulcerative colitis**	55 (0.20%)	235,357	0.23	137 (0.12%)	941,514	0.15	1.61 (1.17–2.20) ^†^	1.56 (1.13–2.15) ^†^

^†^*p* < 0.05 for comparison between patients with two groups. * Each variable was adjusted for gender, age, income, geography, urbanization and comorbidity. PD, periodontal disease; IBD, inflammatory bowel disease. PY, person-years; IR, incidence rate per 1000 person-years; HR, hazard ratio.

**Table 3:** Independent predictors of Ulcerative colitis identified by Cox regression analysis.

Variable		Crude	Adjusted
		HR (95% CI)	HR * (95% CI)
PD		1.61 (1.17–2.20) ^†^	1.56 (1.13–2.15) ^†^
Gender			
	Female	1	1
	Male	1.01 (0.76–1.34)	0.96 (0.72–1.29)
Age			
	20–29	1	1
	30–39	0.47 (0.26–0.86) ^†^	0.46 (0.25–0.85) ^†^
	40–49	0.82 (0.52–1.3)	0.79 (0.49–1.28)
	50–59	0.99 (0.62–1.56)	0.98 (0.6–1.61)
	60–69	1.5 (0.93–2.41)	1.61 (0.95–2.74)
	≥70	1.38 (0.83–2.31)	1.54 (0.87–2.72)
Income			
	<20000	1	1
	20000–39999	0.84 (0.56–1.27)	0.99 (0.65–1.52)
	40000–59999	1.02 (0.61–1.72)	1.26 (0.73–2.17)
	≥60000	2.09 (1.2–3.62) ^†^	2.29 (1.28–4.11) ^†^
Geography			
	North	1	1
	Central	1.79 (1.27–2.51) ^†^	1.86 (1.29–2.66) ^†^
	South	0.87 (0.6–1.25)	0.89 (0.61–1.31)
	Other	2.27 (1.18–4.37) ^†^	2.38 (1.21–4.68) ^†^
Urbanization			
	1 (highest)	1	1
	2	0.77 (0.53–1.11)	0.71 (0.49–1.03)
	3	1.17 (0.82–1.66)	0.99 (0.68–1.45)
	4	0.68 (0.37–1.23)	0.59 (0.31–1.10)
Comorbidity			
	DM	0.95 (0.66–1.37)	0.85 (0.56–1.30)
	Hypertension	1.22 (0.91–1.64)	1.10 (0.75–1.60)
	Hyperlipidemia	0.87 (0.63–1.20)	0.74 (0.50–1.08)
	CAD	1.31 (0.92–1.86)	1.20 (0.80–1.80)
	Stroke	1.32 (0.88–1.98)	1.13 (0.72–1.78)
	Alcoholism	1.00 (0.37–2.69)	1.18 (0.44–3.22)
	Obesity	0.39 (0.05–2.77)	0.45 (0.06–3.24)
	Tobacco use disorder	0.58 (0.34–0.98) ^†^	0.47 (0.27–0.81) ^†^

PD, periodontal disease; DM, Diabetes mellitus; CAD, Coronary artery disease; * Each variable was adjusted for every other variable; ^†^ *p* < 0.05 for comparison between patients with two groups.
